# Associations of activity, sedentary, and sleep behaviors with cognitive and social-emotional health in early childhood

**DOI:** 10.1186/s44167-023-00016-6

**Published:** 2023-04-03

**Authors:** Christine W. St. Laurent, Charlotte Lund Rasmussen, Jennifer F. Holmes, Amanda Cremone-Caira, Laura B. F. Kurdziel, Phillip C. Desrochers, Rebecca M. C. Spencer

**Affiliations:** 1 Department of Psychological and Brain Sciences, University of Massachusetts Amherst, 135 Hicks Way, Tobin Hall, Amherst, MA 01003 USA; 2 Department of Public Health and Nursing, Norwegian University of Science and Technology, Trondheim, Norway; 3 Department of Physical Education and Sport Sciences, University of Limerick, Limerick, Ireland; 4 Faculty of Physical Culture, Palacký University Olomouc, Olomouc, Czech Republic; 5 Department of Psychology, Assumption College, Worcester, MA USA; 6 Department of Psychology, Merrimack College, North Andover, MA USA; 7 Sensing, Perception, and Applied Robotics Division, Charles River Analytics, Cambridge, MA USA; 8 Institute for Applied Life Sciences, University of Massachusetts Amherst, Amherst, MA USA

**Keywords:** Physical activity, Sedentary behavior, Sleep, Cognition, Social-emotional, Children

## Abstract

**Background:**

Early childhood is important for cognitive and social-emotional development, and a time in which to promote healthy movement behaviors (sedentary behavior, physical activity, and sleep). Movement behaviors may have interactive influences on cognition and social-emotional factors in young children, but most previous research has explored them independently. The purpose of this study was to determine if movement behaviors are associated with measures of cognitive and social-emotional health in young children and if so, to describe optimal compositions of movement behaviors of a daily cycle for such outcomes.

**Methods:**

Children (n = 388, 33 to 70 months, 44.6% female) from a clinical trial (ClinicalTrials.gov ID: NCT03285880, first posted September 18, 2017) wore accelerometers on their wrists for 24-h for 9.56 ± 3.3 days. Movement behavior compositions consisted of time spent in sedentary behaviors, light intensity physical activity, moderate to vigorous intensity physical activity (MVPA), and sleep. Outcomes were cognitive (receptive vocabulary, declarative and procedural memory, and executive attention) and social-emotional measures (temperament and behavioral problems). Compositional linear regression models with isometric log ratios were used to investigate the relations between the movement behavior composition and the cognitive and social-emotional health measures. If a significant association was found between the composition and an outcome, we further explored the “optimal” 24-h time-use for said outcome.

**Results:**

Movement behavior compositions were associated with receptive vocabulary. The composition associated with the predicted top five percent of vocabulary scores consisted of 12.1 h of sleep, 4.7 h of sedentary time, 5.6 h of light physical activity, and 1.7 h of MVPA.

**Conclusions:**

While behavior compositions are related to vocabulary ability in early childhood, our findings align with the inconclusiveness of the current evidence regarding other developmental outcomes. Future research exploring activities within these four movement behaviors, that are meaningful to cognitive and social-emotional development, may be warranted.

**Supplementary Information:**

The online version contains supplementary material available at 10.1186/s44167-023-00016-6.

## Background

Early childhood (i.e., under 6 years) is an important life phase in which to promote healthy behaviors of the 24-h cycle (i.e., sedentary behavior, physical activity, and sleep, hereafter collectively referred to as movement behaviors). Specifically, minimizing time spent sedentary, participating in adequate levels of physical activity, and achieving sufficient sleep during childhood can positively impact outcomes such as improved mental and physical health, cognitive performance, and overall quality of life [1–4]. The World Health Organization (WHO) recommends that 4- to 5-year old children be physically active for at least 180 min per day (including at least 60 min of moderate- to vigorous-intensity physical activity [MVPA]), should limit sedentary time (i.e., no more than 60 min of screen time, not be restrained—such as sitting in a stroller or being held—more than 1 h at a time, and avoid sitting for extended periods), and obtain 10 to 13 h of sleep in a 24-h period [5]. Although surveillance data on these health behaviors is limited in younger children, reports indicate that many young children do not obtain sufficient physical activity and sleep, and therefore are likely to engage in more sedentary behaviors [6, 7]. While health promotion regarding these 24-h movement behaviors has traditionally been emphasized in older children, early childhood may be an important time to intervene given that sleep is unique during these early years (as children transition out of naps) and both sleep and physical activity habits track through childhood and even into adulthood [8–10].

Early childhood also serves as an important phase for cognitive development [11, 12]. Cognitive abilities at this life stage underlie both current and future academic performance [13, 14]. In the current study, we focused on the cognitive domains of receptive vocabulary, declarative memory, procedural memory, and executive function (attention). Receptive vocabulary is the ability to understand words and phrases and falls under the umbrella of language skills [15]. Declarative and procedural memory are domains of overall memory that are characterized as long-term memory (e.g., seconds to days) and therefore involves encoding, storage, and retrieval (as opposed to working memory which involves the ability to hold information and manipulate it in the short term) [15]. Declarative memory (i.e., explicit memory) involves the ability to remember experiences, people, and things, whereas procedural memory is a form of implicit memory that allows people to remember motor skills and actions [15–17]. In the preschool years (e.g., 2 years 9 months through 5 years), executive attention is a precursor to executive function (e.g., set shifting, response inhibition, and working memory) and involves the ability to regulate one’s attention during activities where there may conflicting information or stimuli present [18, 19].

Movement behaviors have been independently associated with cognitive outcomes in older children and adults. There is evidence that both acute and chronic physical activity can improve cognitive outcomes such as executive function and memory in these populations [2]. In older adults, physical activity benefits cognitive performance, is associated with a lower risk of dementia, and is beneficial to cognitive impairments in those with dementia [2], while measures of sleep are associated with memory and executive function [20]. There is some evidence of associations of sedentary time and cognitive function in this age group, but findings have been more variable than for physical activity and sleep [21]. Although there has been some support of beneficial associations between physical activity and cognitive measures (and, to some extent, adverse associations with high sedentary time), a lack of sufficient studies limits the conclusions that could be drawn for children under 6 years of age in the 2018 Physical Activity Guidelines Advisory Committee Scientific Report [2]. While a growing number of early childhood studies have been conducted on this topic, recent reviews have indicated that associations between movement behaviors and cognition are more variable than previous reports in older children [22–24]. In contrast, there is accumulating evidence that both daytime and overnight sleep are beneficial for cognitive functions in early childhood [25–28].

Children experience significant social and emotional development in the early years and many measures within this domain have been explored as independent correlates of sedentary time, physical activity, and sleep [29–31]. Broadly, social-emotional health in early childhood encompasses how young children view themselves and their world, their emotions, and their related behaviors. Factors considered in the current paper include: (1) temperament and (2) emotional and behavioral problems. According to Rothbart and Derryberry’s definition, temperament consists of “constitutionally based individual differences in reactivity and self-regulation, influenced over time by heredity and experience” [32]. Common indicators of temperament include surgency (e.g., high positive emotional reactivity levels), negative affectivity (e.g., tendency to experience negative emotional reactivity), and effortful control (e.g., ability to regulate emotions) [33]. Emotional and behavioral problems in young children are commonly categorized as externalizing (e.g., behaviors that are presented and directed outside of the child such as ‘acting out’ and non-compliance) and internalizing problems (e.g., behaviors that are directed within the child such as withdrawing or experiencing anxiety) [34].

In older children, independent associations for sedentary behavior (in the form of screen time), physical activity, and sleep social-emotional measure with social-emotional indicators have been reported [35]. In observational studies where 24-h behaviors have been separately explored with social-emotional outcomes in young children, reported associations have been mixed, particularly for waking behaviors. For example, in a 2017 review, 8 of the 11 included studies employed observational designs to examine physical activity and variables of psychosocial health in children under 5 years. Associations were heterogeneous with favorable, unfavorable, mixed, and null results and quality of evidence designated as “very low [31]”. Comparable findings were noted in a similar 2017 review where sedentary behavior was the exposure of interest (i.e., 9 longitudinal and 7 cross-sectional studies) [29]. In another review of sleep studies in toddlers and preschoolers (5 longitudinal and 17 cross-sectional studies), although shorter sleep duration was more consistently associated with poorer emotional regulation, overall associations were mixed, and the quality of evidence was still designated as “low” [30].

Similar to recent findings in older children, it is possible that movement behaviors may have interactive influences on cognition and social-emotional factors in young children. A number of mechanisms have been proposed or supported in young children connecting sleep (e.g., duration, quality, timing, and routines) with cognitive development and child behavior [25, 28]. For example, longer sleep is often accompanied by greater slow wave sleep, which contains physiological events (e.g., sleep spindles) that are associated with sleep dependent memory consolidation. Additionally, physiological (e.g., changes in brain structure and physiology), psychosocial (e.g., enhanced mood and self-perceptions), and behavioral (e.g., coping and self-regulation skills) pathways have been proposed between physical activity and measures of brain health in children [36]. Sedentary behaviors may potentially play a role in such pathways either by indirect effects on physical activity and sleep, or directly on some mechanisms. Moreover, movement behaviors may influence one another [22, 37, 38] which in turn may facilitate interactive influences on cognitive and social-emotional health [35].

One approach researchers have used to examine interactions of movement behaviors is to explore compliance with guidelines (e.g., meeting national or WHO recommendations for all three 24-h behaviors) as the exposure of interest [35, 39–42]. Another method that has been adopted in recent years by many public health researchers is the use of compositional data analysis [43–45]. Although behavioral data is typically presented as a component of a daily cycle (i.e., min/day or % of day), traditional statistical analysis methods that do not account for the compositional nature of such behaviors (i.e., the co-dependence) are commonly applied. Compositional data analysis can be used to provide information about associations between a behavior of interest and a health outcome while also accounting for time spent in other behaviors, as well as the effect on that relationship when time is reallocated from one behavior to others [46].

Following a trend in preadolescent research, studies have begun to explore relations between movement behaviors and cognitive and social-emotional health with compositional data analysis methods. In one cross-sectional study, theoretical time reallocations replacing sleep or light physical activity with MVPA were associated with improvements in inhibitory control (a measure of executive function) [47]. Another recent study of Canadian preschoolers incorporated measures of the Early Years Toolbox to assess both cognitive (i.e., inhibitory control, visual-spatial working memory) and social-emotional measures (i.e., sociability, externalizing, internalizing, prosocial behavior, and self-regulation) [48]. Overall, sedentary time was positively associated with inhibitory control and vocabulary, and MVPA was positively associated with sociability. When sleep time was theoretically replaced with sedentary time, this was positively associated with vocabulary. Reallocating any behavior in place of MVPA was positively associated with sociability and self-regulation.

Further compositional data analysis studies building on these early reports in young children are necessary to provide greater insight into the interactive relations of 24-h movement behaviors with cognitive and social-emotional health outcomes. Such studies would also determine if previous associations with cognitive and social-emotional health are consistent with different assessments that are commonly utilized by educators and clinicians, as well as with other cognitive and social-emotional health measures (e.g., receptive vocabulary and procedural memory). More recently, given that compositions of behaviors may have differential benefits on various health outcomes, a novel use of compositional data analysis is to explore ‘optimal’ daily combinations of behaviors for different measures [49, 50]. Studies in older children that have used this approach have found variations in best 24-h movement behavior compositions for physical, skeletal, mental, and cognitive health [51–53]. Therefore, the purpose of the present study was to determine if movement behaviors were associated with measures of cognitive and social-emotional health in early childhood. We hypothesized that 24-h movement behavior compositions would be associated with each of the cognitive and social-emotional outcomes while accounting for age and sex. Additionally, we aimed to describe optimal composition of movement behaviors for each of our cognitive and social-emotional measures.

## Methods

### Overview

The data in this study stem from a clinical trial examining whether daytime naps contribute to immediate and delayed benefits on memory in early childhood (ClinicalTrials.gov ID: NCT03285880, first posted on September 18, 2017). The Strengthening the Reporting of Observational Studies in Epidemiology Statement (STROBE) checklist can be viewed in our supplementary materials (see Additional file 1). The study protocol was approved by the University of Massachusetts Amherst Institutional Review Board (approved December 15, 2011; protocol ID: 2011-1152). Written informed consent was obtained from adult caregivers for consent of their own participation as well as permission for their child’s participation. Child participants provided verbal assent and childcare providers provided written informed consent (for completing daytime sleep diaries).

**Fig. 1:**
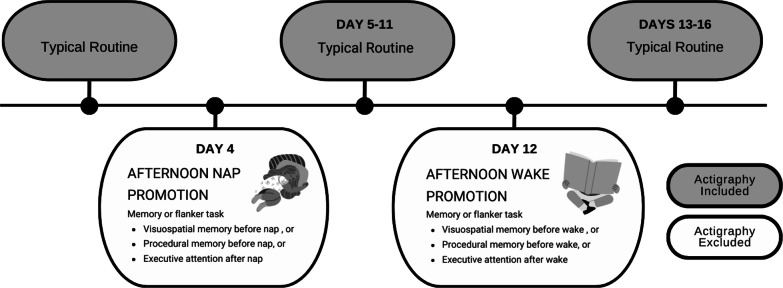
Study overview. Actigraphy was measured throughout the full period, but ‘experimental’ days were excluded because all children were encouraged to either nap or stay awake in the afternoon regardless of their typical routine. Cognitive health measures were collected on nap- and wake-promotion days. Social-behavioral health measures were derived from caregiver/parent complete questionnaires that were distributed on Day 1 and collected at the end of the study period

In brief, the parent study followed a within-subjects design and was conducted over 16 days in preschool and childcare settings (Fig. 1). (See Spencer et al. [54] for more details on the protocol.) At the beginning of the study, adult caregivers completed a questionnaire that included demographics and behavior assessments and children were asked to wear an accelerometer, which was instructed to be worn for the full study period. Participants each completed two conditions (nap- and wake-promotion), 1 week apart in a randomized order. On the nap-promotion day, children were encouraged to nap (e.g., with quiet encouragement and back-rubs) and on the wake-promotion day, the room was dim and quiet, similar to the nap condition, but children participated in sedentary activities such as coloring and reading books. Most participants completed a receptive vocabulary task and at least one other cognitive task (e.g., declarative memory, procedural memory, or executive function).

### Participants

**Fig. 2:**
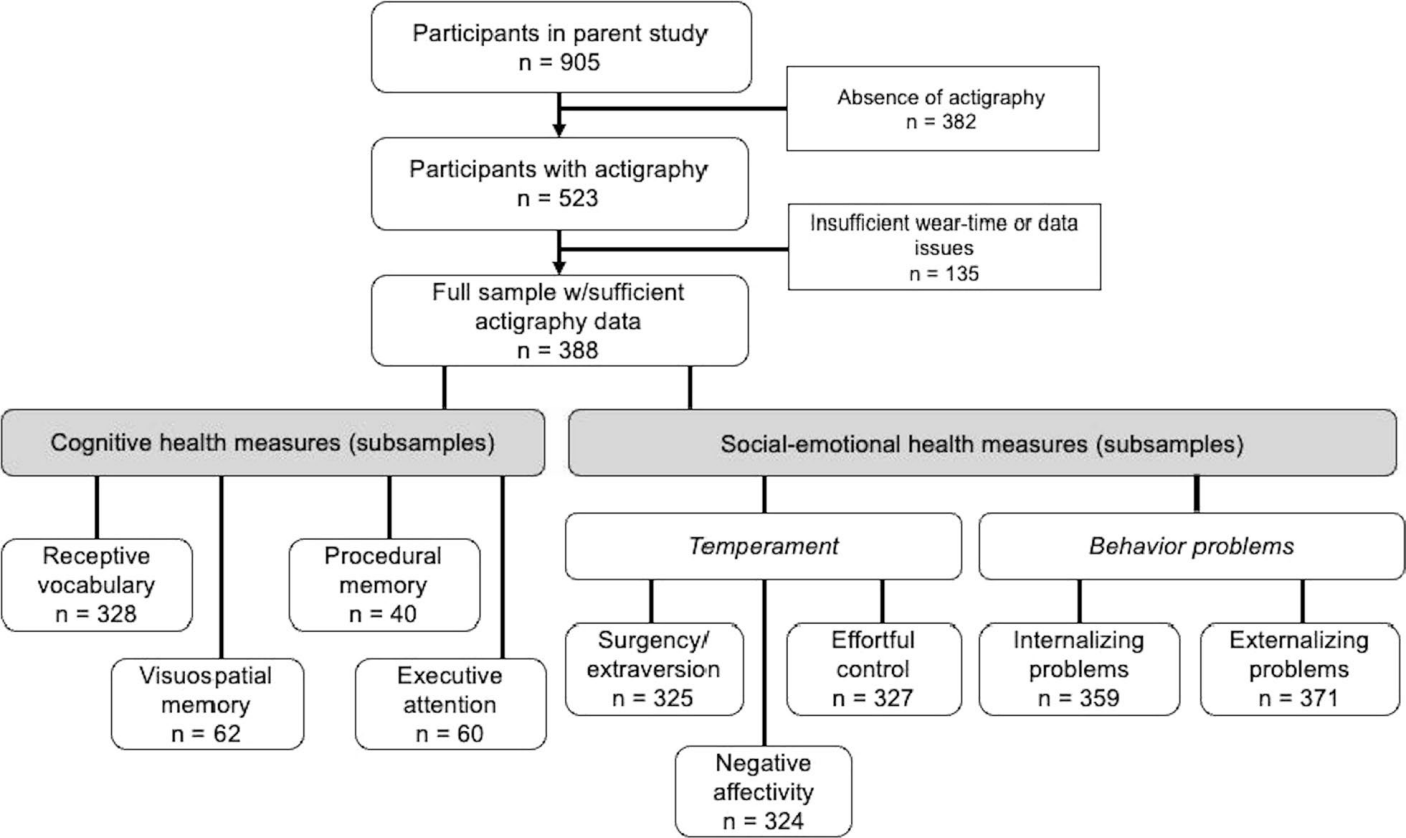
Participant flow diagram and sample sizes of outcome measures. Participant flow diagram and sample size of outcome measures. (For cognitive health, receptive vocabulary was sought for all participants, but each other cognitive task was only completed on a subset of the participants.)

Preschool children were recruited from childcare and preschool centers in western Massachusetts in the United States between 2013 and 2020. To be eligible for the parent study, children: (1) were 33–71 months of age, (2) had normal or corrected-to-normal vision and hearing, (3) had no current or past diagnosis of a developmental disability or sleep disorder, (4) had no use of psychotropic or sleep-affecting medications, and (5) had not recently traveled outside of the local time zone. For the current study, only participants with at least three days and three nights of sufficient actigraphy data (see next section) and at least one of the outcomes of interest were included (Fig. 2).

### 24-h Movement behavior measures

Wake and sleep behaviors were measured with actigraphy. Actiwatch Spectrum monitors were worn on the participants’ non-dominant wrists. The Actiwatch is a triaxial accelerometer with off-wrist detection and a button that can be pressed by participants to mark events. Children were taught how to press the event marker when they began trying to fall asleep and again when they woke. The monitors were configured to collect data in 15-s epochs, with a sensitivity of < 0.01 g and 32 Hz sampling rate. Data was processed in the Actiware software using the default algorithm to designate each epoch categorized as sleep, wake, or off-wrist [55]. This algorithm has demonstrated good agreement with videosomnography in 28- to 73-month-old children and is commonly employed in sleep studies of early childhood [56, 57]. Each daily cycle (i.e., wake onset for day x until wake onset for day x + 1) was then partitioned into overnight rest periods, daytime rest periods (when present), and daytime wake. Therefore, it was possible for averaged daily cycles to fluctuate beyond or below 24 h (or 1440 min). Daily cycles that included an experimental condition (i.e., nap- or wake-promotion) as part of the larger study were excluded as these activities may have differed from a child’s typical routine.

*Sleep.* In children, the Actiwatch is considered an acceptable tool for sleep measurement and has demonstrated validity compared to polysomnography [58, 59]. Rest intervals (i.e., sleep time) for overnight periods and daytime naps (when present) were defined using a combination of marked events (i.e., button presses) and sleep diaries. If neither of these was available, the first three consecutive minutes of sleep (i.e., as categorized by the Actiware algorithm) were used to define sleep onset and the last five consecutive minutes of sleep were defined as sleep offset. For our preliminary models, sleep time was calculated by subtracting wake bouts (i.e., wake after sleep onset and sleep onset latency when diaries were available) from the rest intervals. For our compositional models, the sum of time spent in rest intervals was used as a proxy for sleep time (i.e., we did not exclude wake after sleep onset time).

*Sedentary time and physical activity.* The Actiwatch has been studied for validity and reliability in preadolescent children as an estimate of energy expenditure [60]. Accelerometer activity count cut points were derived from to categorize wake behaviors into sedentary time, light physical activity, and MVPA [60]. These cut points were cross validated in preschool children against direct observation [61]. One recent recommendation of daytime wear time for activity accelerometer estimation in early childhood is a minimum of 600 min [62]. However, as much of our sample received 60 to 120 min of daytime sleep (which was categorized as time in bed rather than wake), days with at least 480 min of daytime wear were included. Therefore, daytime intervals that were defined as wake by Actiware were further processed to estimate sedentary time and physical activity for days that had at least 480 min of actigraphy data. Using the Ekblom et al. [60] cut points, daytime wake intervals were then classified as sedentary time (less than 79 counts), light physical activity (80 to 261 counts), or MVPA (262 counts or greater).

### Cognitive and Social-emotional health measures

#### Cognitive health measures

A detailed description of the cognitive and socio-emotional health measures is provided in Additional file 2. Cognitive measures included receptive vocabulary, visuospatial memory, procedural memory, and executive attention. Receptive vocabulary was evaluated with the Peabody Picture Vocabulary Test, 4th Edition (PPVT-IV) [63] (n = 328), a test that evaluates children’s ability to understand spoken words, and the calculated raw score was used. Reliability of internal consistency of the items has been reported at 0.90 or above across ages groups [63]. Possible scores can range from 2 to 228. This test is not typically used by itself for clinical interpretations regarding vocabulary development, but higher scores represent higher receptive vocabulary and comprehension of spoken English [63].

As an indicator of declarative memory, a visuospatial task similar to the game “Memory” was completed (n = 62). Children were presented with a grid of images on a computer screen and then asked to recall the locations of each. The average immediate accuracy score (for those that scored at least 30%) was used as our declarative memory outcome [17]. Thus, scores could range from 30 to 100%, with a higher score representing better memory performance.

Procedural memory was assessed with a serial reaction time task (n = 41) [64]. In this task, children completed a sequence of finger presses on an electronic tablet and a learning score was calculated as median reaction time in the final sequence blocks minus median reaction time for surrounding random blocks. As such, higher learning scores indicate greater sequence-specific learning [16, 64].

Finally, we evaluated executive attention with a Flanker task (n = 60) similar to a preschool age-adapted version that was used in McDermott et al. [65]. Children were presented with an array of five fish on a computer screen and were instructed to select the direction that the center fish was facing as quickly as possible. Some trials were congruent (i.e., all fish faced the same direction) and some were incongruent (i.e., the outer fish faced the opposite direction of the center fish). We included the accuracy score in our analysis. Higher accuracy scores represented better executive attention performance.

#### Social-emotional health measures

Social-emotional health measures included three sub-scale scores for temperament and two subscales for child behavior. Temperament was explored with scores from the parent-reported Child Behavior Questionnaire Very Short Form (CBQ) [33], with higher scores indicating stronger characteristics reflected by each of the temperament subscales all with a possible score range of 1 to 7: surgency/extraversion (n = 325), negative affectivity (n = 324), and effortful control (n = 327). In children ages 3 to 8 years, internal consistency alpha levels of the Very Short Form ranged between 0.70 and 0.76 for surgency, 0.66 and 0.70 for negative affect, and 0.62 and 0.78 for effortful control [33]. Correspondence corrected correlations between the Very Short Form and the standard Child Behavior Questionnaire were 0.83 for surgency, 0.75 for negative affect, and 0.83 for effortful control [33].

Internalizing and externalizing behavior raw scores (n = 359 and 371, respectively) served as indicators of social and emotional behavior problems from the parent-completed Child Behavioral Checklist for Ages 1.5–5 (CBCL) [66]. Higher scores for both represented that these types of problems were more typical for the child [66]. For clinical purposes, scores under 12 for internalizing problems and under 18 for externalizing problems are often used as cut offs between ‘normal’ ranges and the ‘borderline clinical’ range [66].

### Covariates

Several additional measures were collected to characterize the sample and potentially control for in our models. Age (months), sex, race and ethnicity were obtained from the caregiver questionnaires. Additionally, a composite score for socioeconomic status was calculated using caregiver-reported education, employment status, and household income [67]. Daily total physical activity was parameterized as average activity counts/minute during wake from actigraphy. Actigraphy-measured sleep time (actual estimated time asleep) was also determined for the average daily cycle (i.e., daytime and overnight sleep). Finally, weekly nap frequency (number of days with nap sleep/number of days with usable actigraphy data × 7) was derived from non-experimental days with actigraphy measurement.

### Statistical analyses

Analyses were performed in R version 4.1 [68], using the *compositions* [69] and *car* [70] packages. Standard descriptive statistics (means and standard deviations or as frequencies and percentages) were used to characterize the study population. Preliminary analyses to assess associations between individual movement behaviors, each of the outcomes, and potential covariates were explored with Pearson correlations and multiple linear regression models adjusting for age and sex.

We followed a compositional data analysis (CoDA) approach to describe the children’s 24-h time-use as well as investigate the relations between their movement behaviors and each brain health measure [71]. The approach consisted of several steps. First, for each child, 24-h time-use was defined as a 4-part composition consisting of time spent sleeping, sedentary, in light physical activity, and in MVPA. Compositional means were used to describe the 24-h time-use composition, obtained by calculating the geometric mean of each behavior and then normalizing these to sum to 24 h [71, 72].

Second, compositional linear regression models were used to investigate the relationship between the 24-h movement behavior composition and the cognitive and social-emotional health measures, respectively. This was done by first expressing the 24-h time-use composition as a set of three isometric log-ratio (*ilr)* coordinates [73], which were entered as independent variables in the regression models (i.e., one model per cognitive or social-emotional health outcome). All models were adjusted for age, sex, and grid size/timing (for visuospatial memory only). Multiple regression parameters from the type III analysis of variances were used to assess if the 24-h composition was associated with each brain health measure [74].

Finally, if a significant relationship was found between the 24-h behavior composition and an outcome, we further explored the “optimal” 24-h time-use for said outcome, following the approach described in Dumuid et al. [51]. In short, this was done by predicting cognitive and social health measures for all 24-h compositions represented in the dataset based on the linear regression models. For each outcome measure, the optimal 24-h composition was defined as the means of the compositions associated with the top 5% outcome zone.

## Results

### Participant characteristics

Descriptive characteristics of the 388 preschool-aged participants and sample sizes for each outcome measure are presented in Table 1. Sample sizes were smaller for the memory and executive attention measures due to the protocol of the parent study rather than compliance (i.e., these measures were only collected in subsamples of children). Children ranged in age from 33 to 70 months and our sample had slightly more males than females (55.4% vs. 44.6%). Participants had an average of 739 ± 65.7 min of actigraph wear time during wake intervals and wore the devices for 9.56 ± 3.3 days (range: 3 to 15). The average 24-h movement behavior composition consisted of 5.2 h of sedentary time, 5.3 h of light physical activity, 1.9 h of MVPA, and 11.6 h of sleep. For cognitive health, children’s scores ranged from 10 to 141 for receptive vocabulary performance, 33.0 to 91.7% for visuospatial memory accuracy, − 0.0291 to 0.138.3 for procedural memory learning, and 38.2 to 98.7% executive attention accuracy. For social-emotional health, the range in temperament scores indicated that participants varied in temperament characteristics, and 17% (n = 66) and 17.3% (n = 67) of the sample had a score above ‘normal’ for externalizing and internalizing behaviors respectively, indicating potential behavior concerns for those categories.

**Table 1:** Descriptive characteristics of the study sample

Variables	Mean (SD) or n (%)	Range
Sample characteristics (n = 388)		
Age (months)	51.5 (9.46)	33 to 70
Sex		
Female	173 (44.4)	–
Race		
White	237 (61.1)	–
Black/African American	33 (8.5%)	–
Asian	14 (3.6%)	–
Native Hawaiian/Pacific Islander	2 (0.5%)	–
Two or more racial groups	41 (10.6%)	–
Other	32 (8.2%)	–
Missing	29 (7.5%)	–
Hispanic ethnicity		
Yes	100 (25.8)	
No	268 (69.1)	–
Missing	20 (5.1)	–
Socioeconomic status (score)	4.56 (1.98)	1 to 7
24-h Movement Behaviors (‘absolute’ means)		
Sedentary time (min)	311.7 (72.1)	129.8 to 575.8
Light physical activity (min)	312.5 (41.5)	169.3 to 434.7
MVPA (min)	114.3 (39.7)	18.9 to 292.3
Sleep time (min)	683.6 (45.6)	547.9 to 816.7
Daily cycle weartime (min)	1422.1 (62.3)	1159.0 to 1590.5
24-h Movement Behaviors (compositional means)		
Sedentary time (min)	314.7 (−)	–
Light physical activity (min)	316.3 (−)	–
MVPA (min)	115.8 (−)	–
Sleep time (min)	693.2 (−)	–
Cognitive health		
Receptive vocabulary (PPVT score; n = 328)	86.1 (25.6)	10 to 141
Visuospatial memory accuracy score (%; n = 62)	68.9 (13.7)	33.0 to 91.7
Procedural memory learning score (n = 40)	0.0554 (0.0414)	− 0.0291 to 0.138.3
Executive attention accuracy score (%; n = 60)	65.6 (14.5)	38.2 to 98.7
Social-emotional health		
Surgency/extraversion score (n = 325)	4.49 (0.763)	2 to 6.7
Negative affectivity score (n = 324)	3.75 (0.919)	1.4 to 6.5
Effortful control score (n = 327)	5.00 (0.761)	2.2 to 6.8
Externalizing behavior problem score (n = 359)	8.51 (7.84)	0 to 44
Internalizing behavior problem score (n = 371)	5.11 (5.82)	0 to 52

MVPA: moderate- to vigorous-intensity physical activity

### Preliminary analyses

As a first step in our model building, we explored relations between relevant covariates (i.e., age and sex) with our movement behaviors and brain health outcomes. In respect to movement behaviors, females engaged in more light physical activity and less MVPA than males. Males had higher scores for vocabulary, effortful control, and surgency. There were no differences in age between the females and males. Correlations were explored between age with the movement behaviors and outcomes. As would be expected, age was positively associated with light physical activity, MVPA, vocabulary, and executive attention, and negatively correlated with sleep time.

We next explored preliminary associations between individual absolute movement behaviors (i.e., sedentary time, light physical activity, MVPA, and 24-h sleep time) with each cognitive and social-emotional health outcome. Pairwise unadjusted Pearson correlations demonstrated some significant associations (Additional file 3). Each of the four movement behaviors were correlated with vocabulary development. Specifically, light physical activity and MVPA were positively associated with vocabulary score (*r* = 0.11 and 0.18, respectively) and sedentary time and sleep time were inversely associated (*r* = − 0.18 and − 0.16, respectively). Additionally, MVPA was positively associated with surgency scores (*r* = 0.11), and sleep time was positively associated with procedural memory performance (*r* = 0.32). However, when we next ran multiple linear regression models for each independent movement behavior while adjusting for age and sex (but not other movement behaviors), only a few significant associations were observed (Additional file 4). Light physical activity was positively associated with vocabulary (B = 0.71, *p* = 0.003). Total 24-h sleep time was negatively associated with visuospatial memory (B = − 0.13, *p* = 0.018), but positively associated with procedural memory (B = 0.0003, *p* = 0.038). An interaction of each movement behavior and sex was initially included in each model, but the only significant interaction was between MVPA and sex for negative affectivity, but when stratified by sex, the association did not remain significant. Additionally, including the interaction terms did not improve model fit and therefore were they not included in the reported models.

### Compositional linear regression models

In the compositional data analysis approach, we used compositional linear regression models to investigate the relations between the 24-h movement behavior compositions and the nine cognitive (i.e., receptive vocabulary, visuospatial memory, procedural memory, and executive attention) and social-emotional health outcomes (i.e., three subscales of temperament, and internalizing and externalizing behaviors). Type III analyses of variance F-test were used to determine if the 24-h movement behavior composition was associated with each of the outcomes (Table 2). The 24-h time use composition of our preschool participants was only associated with receptive vocabulary (F = 5.242, *p* = 0.002).

**Table 2:** Associations between overall 24-h movement behavior composition and cognitive/social-emotional health measures

	*df*	F-value	*p*-value
Cognitive health			
Receptive vocabulary	3	5.242	0.002
Visuospatial memory	3	0.3573	0.784
Procedural memory	3	0.131	0.941
Executive attention	3	0.465	0.708
Social-emotional health			
Surgency	3	1.252	0.291
Negative affectivity	3	0.025	0.995
Effortful control	3	0.490	0.690
Externalizing behavior	3	0.195	0.900
Internalizing behavior	3	0.035	0.991

All models included the four 24-h movement behaviors expressed as three isometric log-ratio coordinates and were adjusted for age and sex. *The model for visuospatial memory was also adjusted for grid size and timing

### Optimal time-use for receptive vocabulary

Given the significant association between the movement behavior composition and receptive vocabulary, we further explored the ‘optimal’ 24-h time-use for that specific cognitive health measure. The 24-h composition associated with the 5% best vocabulary scores (i.e., raw scores of 122 to 141) consisted of 12.1 h of sleep, 4.7 h of sedentary time, 5.6 h of light physical activity and 1.7 h of MVPA (Table 3). The top 5% represented 23% of our sample (n = 77 out of 328 children).

**Table 3:** Optimal 24-h movement behavior composition for vocabulary score

Compositional mean, hours/day [min; max]	
Sleep time	12.1 [10.2; 15.5]
Sedentary time	4.7 [2.3; 9.1]
Light physical activity	5.6 [3.8; 7.0]
MVPA	1.7 [0.3; 4.8]

## Discussion

In this study, among the nine cognitive and social-emotional health measures that we examined, the daily time use composition of movement behaviors was only associated with receptive vocabulary in our early childhood sample. A daily profile that is similar to current WHO recommendations for physical activity and sleep in preschool aged children [5] corresponded to the top five percent of receptive vocabulary scores (i.e., 12.1 h of sleep, 4.7 h of sedentary time, 5.6 h of light physical activity, and 1.7 h of MVPA). These findings align with the inconclusiveness of the current evidence regarding the relations between daily movement behaviors and similar components of brain health in early childhood.

The sole significant association in the current study for receptive vocabulary was particularly interesting as PPVT scores may be a proxy for IQ, which is reflective of brain development in the early years [26, 75]. Although early childhood vocabulary ability has been minimally explored in studies using compositional data analysis, one study had complementary findings for this cognitive domain [48]. Kuzik et al. [48] reported an association between the 24-h behavior composition of their preschool sample and expressive vocabulary. While accounting for the other behaviors, sedentary time (referred to as stationary time in their report) was positively associated with vocabulary. Reallocating time from sleep to sedentary time was associated with estimated increases in vocabulary. As they measured expressive vocabulary with a different tool (i.e., the Early Years Toolbox), it is notable that between the two studies movement behaviors were related to both expressive and receptive vocabulary. While early findings show agreement, this domain should be further explored in young children to see if results are generalizable across populations and assessment methods. While speculative, it is possible that children who are more active may have more opportunities to interact with others and thus practice and develop their vocabulary skills. Furthermore, physical activity may contribute to better sleep (e.g., duration and quality) which in turn is associated with vocabulary development [76, 77]. Thus, exploring temporality of these behaviors and vocabulary outcomes, as well as exploring contexts and specific activities within the movement behaviors, would provide greater insight into this association.

Movement behavior compositions were not associated with either of our memory outcomes or executive attention, impeding our ability to determine optimal compositions for these measures. In contrast to our finding, Kuzik et al. [48] reported that memory performance was associated with movement behavior compositions with a similar visuospatial task, albeit to assess working memory rather than declarative memory as in the current study. To our knowledge no other study has explored declarative memory outcomes in early childhood samples. Interestingly, compositional data analyses of movement behaviors and memory in older children have also been scarce and limited to working memory measures [78]. The lack of associations is somewhat surprising as reports of physical activity and, more consistently, sleep have been correlated to memory performance when explored independently [79, 80]. An important consideration to the current approach measure of sleep time in the compositional models did not exclude wake bouts while in bed. Indeed, actual sleep time, rather than time in bed (or in the present study total time in rest intervals), may be more influential on memory [80]. Given that healthy sleepers of this age have generally low time spent in wake after sleep onset [81], this is not likely a substantial concern. However, in addition to investigating memory domains outside of working memory in future work, exploring subcomponents of sleep (e.g., sleep onset latency, sleep duration, and wake after sleep onset) may be warranted.

While evidence regarding associations of movement behavior compositions and executive functions is also limited, one study in older children suggested favorable associations with some indicators of executive function [78], whereas findings in younger children have been mixed. In a study in Brazilian preschoolers, the movement behavior composition was associated with inhibitory control [47]. When time was reallocated from sleep or light physical activity to MVPA, this corresponded with estimated improvements in inhibitory control. However, comparable to our findings, Kuzik et al. [48] reported no association between the movement behavior composition and response inhibition. Interestingly, these two comparison studies both used the Go/No-Go task from the Early Years Toolbox, but had conflicting results.

Differences in sample movement behavior compositions could possibly contribute to this discrepancy. The time-use composition in the current study is similar to the behavior profile in Kuzik et al. [48] (i.e., 6.05 h sedentary time, 5.09 h of light physical activity, 1.75 h of MVPA, and 11.2 h of sleep), whereas Bezzera’s composition had greater levels of sedentary time and lower levels of MVPA (7.6 h of sedentary time, 4.2 h of light physical activity, 0.84 h of MVPA, 11.4 h of sleep). Given that socioeconomic status is often inversely associated with both physical activity and sleep of children, differences in compositions could be related to social-demographic differences of the sample given that Bezzara et al. [47] studied children from families that reported lower socioeconomic status. Additionally, the sample in the Bezerra et al. study lived in a middle-income country, as opposed to the high-income countries sampled for Kuzik et al. and the present study (Canada and the United States), and environmental differences may also contribute to the associations between movement behaviors and the outcomes.

Also similar to memory, early childhood studies exploring behavior compositions with social-emotional measures are limited and like the current findings, generally null. For example, Kuzik et al. [48] explored numerous subscale scores of the Child Self-Regulation and Behaviour Questionnaire (i.e., behavioral self-regulation, cognitive self-regulation, emotional self-regulation, externalizing, internalizing, sociability, and prosocial behavior). Although some time reallocations were significantly associated with estimated changes in some outcome measures, movement behavior compositions were not significant with any of these scores. In the current study we explored social-emotional variables as separate outcomes. However, one consideration for future work is that the relation between sleep and emotional regulation may be moderated by temperament [82, 83]. Additionally, it is important to further breakdown components of wake behaviors to consider contexts and modalities that may be relevant to social-emotional development.

Although movement behavior composition studies are also somewhat limited in older children, there appears to be some emerging support of an association between behaviors and social-emotional outcomes for preadolescents. For example, in one cross-sectional analysis, the time-use composition of 10- to 12-year-old Australian children was associated with internalizing behaviors and total difficulties scores [84]. Specifically, in relation to other behaviors, sleep was negatively associated with internalizing problems and total difficulty scores, sedentary time was positively associated with internalizing problems, and light physical activity was positively associated with internalizing problems and total difficulties scores. Another study in 9- to 13-year-old British children noted that the sample’s movement behavior composition was associated with internalizing problems and prosocial behavior, but only in primary school students. Specifically, sedentary time was positively associated with internalizing problems and negatively associated prosocial behavior. In the current analysis, we may not have a generalizable range of child behavior scores (e.g., children generally had low behavioral problem scores), which could in turn influence our findings. This could be related to both the inclusion criteria of the parent study (i.e., no diagnosed sleep disorders or developmental disabilities) and the possibility of participation bias (e.g., families with children presenting more behavior challenges may be less likely to enroll in the study).

While the current study examined a range of cognitive and social-emotional measures, some considerations should be taken into account. In addition to the potential limitations noted for behavioral measures, current findings may not be generalizable to other early childhood populations given the general healthy characteristics of our sample. As with other accelerometry devices, there is room for misclassification of behaviors given different data configuration and processing protocols, and that the waist is generally preferable for physical activity metrics [85], whereas the wrist is recommended for sleep [86]. However, wearing one device generally leads to better compliance [62] and the time spent in different behaviors of the present study are comparable to those in Kuzik et al.’s [48] sample of Canadian preschool children. Additionally, it may be that our behavioral composition components were too ‘broad’ for these cognitive and social-emotional outcomes, and future researchers could look at more nuanced measures (i.e., subcomponents of behaviors). For example, reading and traditional learning activities that my help with cognitive performance are most likely to consist of sedentary behaviors, and some types of physical activity may be more beneficial than others (e.g., cognitively engaging or incorporating executive functions skills) [87]. Dose, modality, intensity, and timing could all play a role here, but our data could not tease apart those potential moderators. Finally, we were unable to compare ‘best’ compositions across outcomes as some measures were only collected on a subgroup of participants.

## Conclusions

In this sample of preschool aged children, 24-h movement behavior compositions of sedentary time, light physical activity, MVPA, and sleep were generally not associated with cognitive and social-emotional health outcomes. However, consistent with other studies, the time-use of these behaviors does appear to be related to vocabulary knowledge. While the present findings are generally in alignment with other early childhood reports that utilized compositional data analysis with the same four movement behaviors and mental health related outcomes, future work should consider activities within the broad behaviors—as they may be more meaningful to such outcomes—in more health diverse samples.

### Supplementary Information

**Additional file 1. **STROBE statement cross-sectional study checklist.

**Additional file 2. **Detailed methods for the cognitive and social-emotional health assessments

**Additional file 3. **Pairwise correlations between absolute movement behaviors and cognitive/social-emotional health outcomes.

**Additional file 4. **Associations between absolute movement behaviors and cognitive/social-emotional health outcomes with linear regression models.

## Data Availability

The datasets used and/or analyzed during the current study are available from the corresponding author on reasonable request.

## Abbreviations

CBCL: Child Behavioral Checklist for Ages 1.5–5
CBQ: Questionnaire Very Short Form
MVPA: Moderate to vigorous intensity physical activity
