# Supplementary material for: Associations of activity, sedentary, and sleep behaviors with cognitive and social-emotional health in early childhood
Source: J Act Sedentary Sleep Behav. 2023 Apr 3;2:7. doi: 10.1186/s44167-023-00016-6 (PMC11116218; doi:10.1186/s44167-023-00016-6)
Supplement: Supplementary file 2 — Additional file 2. Detailed methods for the cognitive and social-emotional health assessments [file 44167_2023_16_MOESM2_ESM.pdf]

Additional File 2. Detailed methods for the cognitive and social-emotional health assessments.

Cognitive Health Measures

Receptive vocabulary. Receptive vocabulary was evaluated with Peabody Picture Vocabulary Test, 4th Edition (PPVT-IV) (1), typically during the morning or during the afternoon of the wake-condition of the clinical trial. The PPVT in a standardized vocabulary test that is commonly used to assess the understanding of spoken American English words (2). Children (n = 328) were presented four images at a time and asked to point to the picture that corresponded with a verbal statement made by the researcher (e.g., "Show me painting"). When a child correctly identified eight items, this established the "basal" score. A score of one was given if the child was not successful. A "ceiling" level was set as the point when a child incorrectly identified six of eight consecutive items. Raw scores were calculated by adding the number of correct responses between the basal score and ceiling level to the basal score.

Visuospatial memory. As an indicator of declarative memory, a visuospatial task similar to the game "Memory" was completed in a subsample of participants (n = 62). This task was administered on a computer or electronic tablet. Children were presented with a grid of either 12 or 16 images (i.e., items). During the encoding phase, children were asked to identify each item. Then, images were 'flipped over' (i.e., replaced with a blue square) and children were asked to point to the location of various images on the grid as each item was presented one at a time. Feedback was provided during this phase, and it continued until the participant achieved 75% accuracy. The immediate recall phase followed the encoding phase. During this phase children were asked to point to the location of each item without feedback. Immediate recall accuracy was calculated as the number of items correctly recalled divided by the number of items presented. Participants completed the task on the two experimental condition days either before nap- or wake-promotion (n = 39) or after (approximately 30 minutes; n = 23) nap- or wake-

promotion. The immediate memory accuracy score was averaged for the two days. To include children that were attending to the task, we only included those with average accuracy scores over 30%. When participants only completed the task for one condition (n = 8), the accuracy score for that day was used.

Procedural memory. A serial reaction time task (3) delivered on an electronic tablet and adapted by our research group for preschool children was used to assess procedural memory (4). In this task, children (n = 41) were instructed to complete a sequences of finger presses on virtual colored “buttons” presented on the tablet screen. For each sequence, the image of a cartoon dog would appear on one button at a time and children were instructed to “catch the dog” by pressing the corresponding button as quickly as possible with their index finger of their non-dominant hand without pressing the wrong button. First, children completed a training phase that consisted of seven blocks. In each block, the same five-item sequence was repeated eight times. The immediate test phase occurring directly after training and consisted of three blocks. In the first and third block, the previously learned sequence was repeated eight times each. The second block consisted of 40 cues that were in a semi-random order. Procedural memory was expressed as two variables, a learning score and reaction time. To calculate a learning score, first reaction time for each trial was determined as the time between stimulus presentation and subsequent button touch. Within each block, if an individual trial’s reaction time was above or below two standard deviations of that block (e.g., could be due to loss of attention), that trial was excluded. For the remaining trials within each block, an accuracy score consisted of the number of correct trials divided by the number of total trials and a median reaction time was determined for each block. Finally, the learning score was calculated as the median reaction time for the random block (i.e., block 2) minus the average of the median reaction times for the sequence blocks (blocks 1 and 3) from the immediate test phase (5) Learning scores that were below or

above two standard deviations from the learning score mean were excluded. Reaction time was the median reaction time of blocks 1 and 3 of the immediate test phase. This task was administered in the morning of the two experimental conditions and therefore the reaction times and learning scores for the two days were averaged. However, if a participant was missing data for one condition (n = 7), the learning score was used for one day of data.

Executive attention. Executive attention was assessed with a Flanker task similar to one used by McDermott et al. (6) in young children. Stimuli were five fish presented on a computer screen with the center fish as the target fish. A trial was "congruent" if the flanking fish were facing the same direction as the target fish or "incongruent" if the flanking fish were pointing in the opposite direction of the target fish. Children completed a practice block of 10 trials and two experimental blocks of 40 trials each. Each block had an equal number of congruent and incongruent trials that were presented in a randomized order. For each trial, first a fixation mark would appear on the center of the screen followed by the fish stimuli. Children were instructed to click on the side of the computer mouse that corresponded to the direction that the target fish was facing (i.e., left of right) as quickly and accurately as possible for each trial. Feedback was provided for each response. A smiley face would appear if the response was correct, and a frown face was presented if the response was incorrect. If children did not respond within 1,300 ms, that trial was omitted. The accuracy score (%: the number of correct trials divided by the total number of trials) was used as the executive attention variable. This task was completed on the experimental days after the nap- or wake-condition and the scores were averaged between days.

Social-Emotional Health Measures

Temperament. Temperament was assessed with the parent-reported Child Behavior Questionnaire Very Short Form (CBQ) (7). The CBQ consists of 36 items that assess three broad scales. Parent-reported subscale scores of children ages 3 to 8 years for the CBQ Very Short Form correlate significantly with scores from the full CBQ (7,8). In the present study, parents rated each child participant's level of emotional reactivity for each item. Ratings were selected from a 7-point Likert scale that ranged from 1 ("Extremely Not True") to 7 ("Extremely True"). "Not applicable" was also an option choice for each item. Subscale scores were calculated for surgency/extraversion (average of 12 items relating to impulsivity, shyness, activity level, and high intensity pleasure), negative affectivity (average of the 12 items relating to anger, discomfort, sadness, soothability, and fear), and effortful control (average of 12 items relating to inhibitory control, attention focusing, low intensity pleasure, and perceptual sensitivity). Higher scores reflect stronger characteristics reflected by each of the temperament subscales.

Social and emotional behavior problems. Internalizing and externalizing behavior scores served as indicators of social and emotional behavior problems. Parents and teachers completed the Child Behavioral Checklist for Ages 1.5-5 (CBCL), which has demonstrated to be reliable for early childhood samples (9). The CBCL consists of 100 statements and parents were asked to select the response that best reflected the child's typical behavior with a 3-point Likert scale (0 = "Not True", 1 = "Somewhat or Sometimes True", and 2 = "Very True or Often True"). The internalizing problem score was the sum of responses to subscale scores assessed typical behaviors relating to emotionally activity, anxiety and depression, somatic complaints, and withdrawn behaviors. The externalizing score was the sum of responses to subscale scores regarding attention problems and aggressive behaviors. Higher scores for both represented that these types of problems were more typical for the child.

References

1. Dunn LM, Dunn DM, Bulheller S. Peabody Picture Vocabulary Test: PPVT. Swets Test Services; 2003.
2. Carvajal, H. H, Parks JP, Logan RA, Page GL. Comparisons of the IQ and vocabulary scores on Wechsler Preschool and Primary Scale of Intelligence-Revised and Peabody Picture Vocabulary Test-Revised. Psychol Sch. 1992;29(January):22–4.
3. Savion-Lemieux T, Bailey JA, Penhune VB. Developmental contributions to motor sequence learning. Exp Brain Res. 2009;195(2):293–306.
4. Desrochers P, Kurdziel LBF, Spencer RMC. Delayed benefit of naps on motor learning in preschool children. Exp Brain Res. 2016;234(3):763–72.
5. Nissen MJ, Bullemer P. Attentional requirements of learning: evidence from performance measures. Cogn Psychol. 1987 Jan;19:1–32.
6. McDermott JM, Pérez-Edgar K, Fox NA. Variations of the flanker paradigm: Assessing selective attention in young children. Behav Res Methods. 2007;39(1):62–70.
7. Putnam SP, Rothbart MK. Development of short and very short forms of the children’s behavior questionnaire. J Pers Assess. 2006;87(1):102–12.
8. Rothbart MK, Ahadi SA, Hershey KL, Fisher P. Investigations of Temperament at Three to Seven Years: The Children’s Behavior Questionnaire. Child Dev. 2001;72(5):1394–408.
9. Achenbach TM, Ruffle TM. The Child Behavior Checklist and Related Forms for Assessing Behavioral/Emotional Problems and Competencies. Pediatr Rev. 2000;21(1):265–71.
