# Supplementary material for: Associations of activity, sedentary, and sleep behaviors with cognitive and social-emotional health in early childhood
Source: J Act Sedentary Sleep Behav. 2023 Apr 3;2:7. doi: 10.1186/s44167-023-00016-6 (PMC11116218; doi:10.1186/s44167-023-00016-6)
Supplement: Supplementary file 3 — Additional file 3. Pairwise correlations between absolute movement behaviors and cognitive/social-emotional health outcomes. [file 44167_2023_16_MOESM3_ESM.pdf]

Additional File 3. Pairwise correlations between absolute movement behaviors and cognitive/social-emotional health outcomes.

Variables	(1)	(2)	(3)	(4)
(1) Sedentary time	1.000			
(2) Light PA	-0.770*	1.000		
	(0.000)			
(3) MVPA	-0.789*	0.246*	1.000	
	(0.000)	(0.000)		
(4) 24-hr sleep	0.078	0.026	-0.159*	1.000
	(0.123)	(0.614)	(0.002)	
(5) Vocabulary	-0.178*	0.113*	0.180*	-0.163*
	(0.001)	(0.041)	(0.001)	(0.003)
(6) Internalizing behavior	-0.010	0.021	-0.003	-0.002
	(0.847)	(0.686)	(0.961)	(0.968)
(7) Externalizing behavior	-0.019	-0.007	0.035	0.026
	(0.719)	(0.898)	(0.500)	(0.620)
(8) Surgency	-0.008	-0.107	0.110*	-0.058
	(0.884)	(0.055)	(0.048)	(0.299)
(9) Negative affectivity	-0.018	0.007	0.027	-0.058
	(0.740)	(0.904)	(0.627)	(0.302)
(10) Effortful control	0.013	0.047	-0.063	-0.074
	(0.812)	(0.400)	(0.257)	(0.180)
(11) Visuospatial memory	-0.092	0.102	0.047	-0.202
	(0.478)	(0.429)	(0.717)	(0.116)
(12) Executive attention	-0.146	0.242	0.025	-0.052
	(0.266)	(0.063)	(0.847)	(0.694)
(13) Procedural memory	-0.093	0.024	0.136	0.321*
	(0.567)	(0.885)	(0.404)	(0.043)

* $p<0.05$ (PA = physical activity; MVPA = moderate to vigorous intensity physical activity)
