# Supplementary material for: Associations of activity, sedentary, and sleep behaviors with cognitive and social-emotional health in early childhood
Source: J Act Sedentary Sleep Behav. 2023 Apr 3;2:7. doi: 10.1186/s44167-023-00016-6 (PMC11116218; doi:10.1186/s44167-023-00016-6)
Supplement: Supplementary file 4 — Additional file 4. Associations between absolute movement behaviors and cognitive/social-emotional health outcomes with linear regression models. [file 44167_2023_16_MOESM4_ESM.pdf]

Additional File 4. Associations between absolute movement behaviors and cognitive/social-emotional health outcomes with linear regression models.

Outcomes	Sedentary time<br>Coef.<br>(95% CI)	Light PA<br>Coef.<br>(95% CI)	MVPA<br>Coef.<br>(95% CI)	24-hr sleep<br>Coef.<br>(95% CI)
Vocabulary	-.27<br>(-.55 to .02)	.71*<br>(.25 to 1.18)	.07<br>(-.40 to .54)	-.01<br>(-.08 to .05)
Internalizing behavior	-.01<br>(-.09 to .06)	.02<br>(-.11 to .14)	.02<br>(-.11 to .14)	-.002<br>(-.02 to .01)
Externalizing behavior	-.02<br>(-.12 to .08)	.0009<br>(-.16 to .17)	.04<br>(-12 to .21)	.005<br>(-.02 to .03)
Surgency	.003<br>(-.008 to .013)	-.01<br>(-.03 to .006)	.004<br>(-.01 to .02)	-.0004<br>(.002 to .002)
Negative affectivity	-.0009<br>(-.01 to .01)	.00005<br>(-.02 to .02)	.003<br>(-.02 to .02)	.0002<br>(-.0008 to .001)
Effortful control	.0003<br>(-.01 to .01)	.003<br>(-.01 to .02)	-.004<br>(-.02 to .01)	-.002<br>(-.003 to .0004)
Visuospatial memory	-.08<br>(-.62 to .46)	-.32<br>(-1.2 to .60)	.54<br>(-.36 to 1.42)	-.13*<br>(-.24 to -.02)
Executive attention	-.16<br>(-.52 to .19)	.32<br>(-.32 to .95)	.14<br>(-.41 to .68)	.03<br>(-.05 to .11)
Procedural memory	-.0001<br>(-.001 to .002)	.0005<br>(-.002 to .003)	-.0004<br>(-.003 to .06)	.0003*<br>(.00002 to .00006)

*p < .05 (Coef = regression coefficient; CI = confidence interval; PA = physical activity; MVPA = moderate to vigorous intensity physical activity) Note: All models were adjusted for age and sex. Models for vocabulary were also adjusted for grid size and timing.
